# High‐Throughput Mechanical Rupture of Nuclear Envelope and the Intracellular Dynamics of Massive Wound Repair

**DOI:** 10.1002/advs.76561

**Published:** 2026-07-14

**Authors:** Apresio K. Fajrial, Leyla Akh, Stephanie E. Schneider, Wei Tan, Corey P. Neu, Xiaoyun Ding

**Affiliations:** ^1^ Department of Mechanical Engineering University of Colorado Boulder Colorado USA; ^2^ Biomedical Engineering Program University of Colorado Boulder Colorado USA; ^3^ BioFrontiers Institute University of Colorado Boulder Colorado USA; ^4^ Material Science and Engineering Program

**Keywords:** cell membrane, nuclear envelope, rupture, wound repair

## Abstract

The dynamics of nuclear envelope rupture and wound repair are critical biological processes that play essential roles in cell homeostasis. Previous studies on nuclear envelope repair dynamics have mainly focused on small ruptures induced by low‐throughput tools such as laser ablation and atomic force microscopy. Here, a device is presented that deterministically porates the cell membrane and nuclear envelope in high throughput, with applications in single‐cell studies of wound repair dynamics and in statistical assessments of cell poration. The device consists of sharp nanostructures in arrays of microchannels, enabling precise and localized disruption at both the plasma membrane and nuclear envelope while preserving cell viability. The distribution of endosomal sorting complexes required for transport (ESCRT) proteins after mechanical disruption at both the plasma membrane and nuclear envelope highlights the cell recovery strategy for severe wounds. Interestingly, during this extreme rupture, resources seem to be allocated more toward nuclear envelope repair. High‐throughput mechanoporation on membrane systems provides a new perspective on studying complex wound repair dynamics, opening promising avenues for further research in the field of wound healing mechanisms.

## Graphical abstract

A microfluidic device with monolithically integrated nanostructures enables high‐throughput wounding of the cell membrane and nuclear envelope. The spatial distribution of charged multivesicular body protein 4B (CHMP4B) within the cell is mapped before and after wounding, showing involvement in repair of both the cell membrane and nuclear envelope. Greater resources are allocated toward nuclear envelope repair than cell membrane repair.

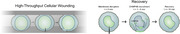

## 1 Introduction

The nuclear envelope of the cell acts to protect and regulate the stability and expression of genomic DNA [1, 2, 3]. As such, significant correlations between nuclear envelope integrity and cell and human health have been found, including in cancers, myopathies, and neuropathies [1, 2, 4, 5, 6]. Malignant cancers show significant changes to nuclear morphology and nuclear envelope function with correlations to the metastasis process, wherein tumor cells squeeze through narrow constrictions to enter and exit vasculature [7, 8, 9]. Damage to the cell membrane and nuclear envelope, whether induced by injury or cell migration, initiates a cascade of membrane repair responses involving calcium influx, annexins, and more recently reported, the endosomal sorting complexes required for transport (ESCRT) machinery [10]. The ESCRT membrane repair mechanism has also been implicated in enabling tumor cell evasion from cytotoxic T‐cell attack [11]. Understanding cell recovery after membrane damage is therefore critical to understanding tumor cell survival, metastatic potential, and for the development of therapies that target membrane repair pathways.

ESCRT‐mediated repair of the plasma membrane and nuclear envelope is well established [9, 12], and defects of ESCRT protein machinery can lead to cancers and severe neurological pathologies [13]. During cellular wound repair, ESCRT is recruited to the site of membrane damage and acts to repair membranes within minutes [12]. However, existing studies of ESCRT recruitment have primarily characterized responses to small, single‐membrane wounds generated by laser ablation or narrow cell‐confining constrictions [9, 12]. The response of ESCRT machinery to large wounds, and repair resource allocation during simultaneous wounding of the nuclear envelope and plasma membrane, remain poorly characterized. These responses are directly relevant to tumor cell survival during metastatic migration, where cells experience simultaneous and severe deformation of both membranes, and may open avenues for therapeutic interventions that interrupt membrane repair pathways.

Existing tools to study plasma membrane and nuclear envelope repair pathways include micropipette‐ and atomic force microscopy (AFM)‐based wounding, as well as cell migration through narrow constrictions that model confined tumor cell migration [9, 12]. These approaches allow for precise time‐resolved analysis of single‐cell responses to damage but are limited in two respects: they generate small wounds that do not represent severe mechanical disruption, and their single‐cell nature cannot wound large groups of cells in synchrony to study population‐level repair dynamics. High‐throughput and single‐cell temporal resolution represent a tradeoff in the field of cell wounding studies, and tools for high‐throughput wound generation and recovery analysis on entire populations of cells remain rare [9, 12, 14, 15, 16].

To address this gap, we used the Nano‐Engineered Surface Technology (NEST) device, a microfluidic platform with integrated monolithic nanostructures previously reported for high‐throughput intracellular delivery [17]. The NEST device enables single‐point simultaneous disruption of both the plasma membrane and nuclear envelope and produces large ruptures in a size regime that has not been characterized using existing technologies (in the range of 7 µm on the plasma membrane and 2 µm on the nuclear envelope). The high‐throughput capability of this device enables synchronous wounding of cell populations, as well as subsequent statistical assays and time‐resolved single‐cell analysis of repair dynamics from a synchronized population. Cell viability is also preserved despite severe wound generation, enabling downstream biological studies. The combination of large, controlled wound generation with high‐throughput capabilities while preserving cell viability enables investigation of ESCRT repair dynamics in an understudied wound severity regime with broad implications for understanding membrane repair.

In this study, we first characterize the cellular wounds generated by the NEST device and confirm high‐throughput wounding of cell populations. Then, we perform single‐cell CHMP4B distribution analysis at defined time points after population‐scale wounding. We find that under severe mechanical disruption of plasma membranes and nuclear envelopes, CHMP4B is massively mobilized from the nucleus to the sites of membrane damage, with preferential allocation of resources toward repair of the nuclear envelope. Our results provide a new perspective on resource allocation and repair hierarchy under extreme mechanical wounds with implications to our understanding of fundamental biology and human disease, including cancer and laminopathies.

## 2 Results

### 2.1 Design and Characterization of the Nanostructured Microfluidic Device

**FIGURE 1:**
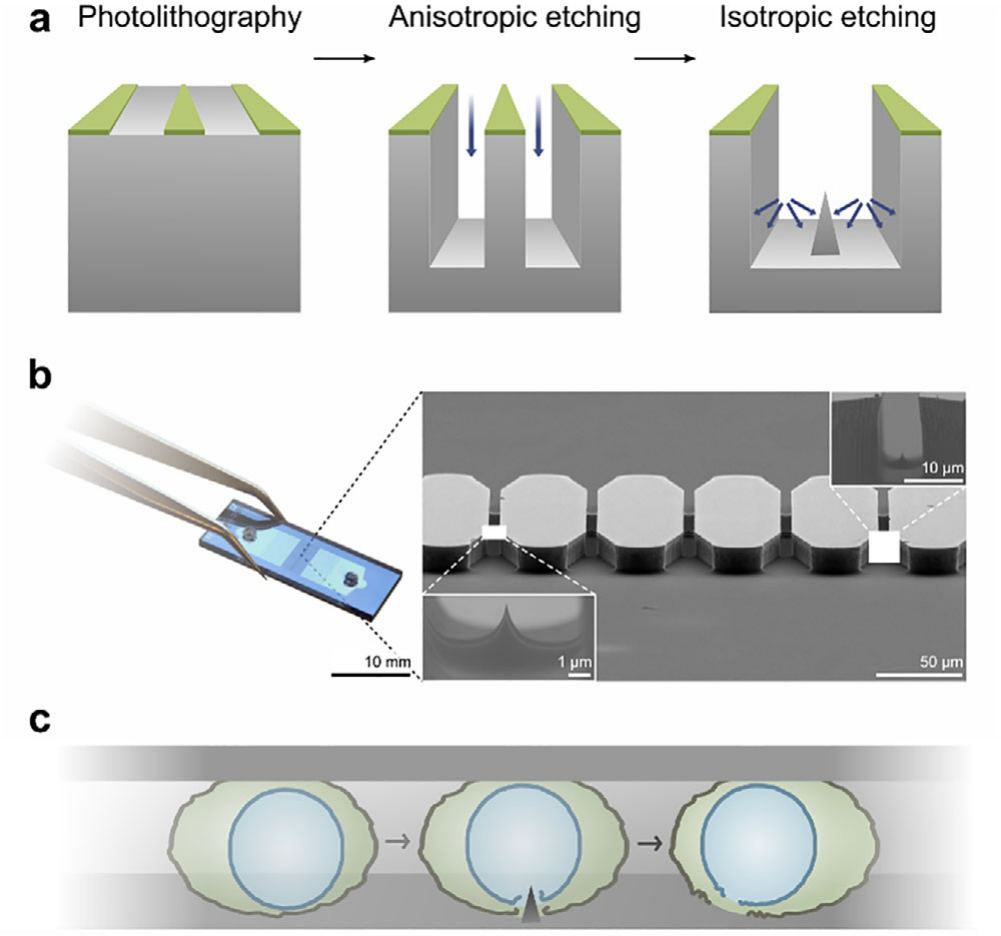
Design principles of the Nano‐Engineered Surface Technology (NEST) device and characteristics of permeabilization. (A), Microfabrication strategy to create the NEST device that consists of photolithography on a silicon wafer followed by a combination of anisotropic and isotropic etching. (B), Representative photograph of a fabricated silicon‐glass NEST device held by tweezers. Inset shows tilted angle scanning electron micrograph of the NEST device. (C), Schematic demonstration of the membrane and nuclear envelope disruption method of the NEST device, as single cells flow through a channel with a monolithically integrated nanostructure.

High‐aspect ratio and sharp‐tip nanostructures are an effective strategy for creating cell membrane openings, as reported in previous studies [17, 18, 19, 20]. To enable high‐throughput cell processing, we developed the Nano‐Engineered Surface Technology (NEST) device, which integrates nanostructures into a continuous flow microfluidic system (Figure 1a) [17]. Sharp protruding nanostructures (nanolancets) are added to the microchannels to allow deterministic permeabilization of the cells. The nanolancets are designed to create a precise incision up to the nuclear envelope surface while preserving cell viability (Figure 1b).

To ensure flexibility in fabrication procedures and retain the potential for high‐volume manufacturing using conventional semiconductor processing, we used silicon wafers as the main substrate and employed standard microfabrication processes. The fabrication strategy of the NEST device involves three main steps (Figure 1a). First, microscale patterns are created using photolithography. Second, microchannels are etched using anisotropic etching to create a trench. The geometry of the microchannels is essential to ensure proper contact between cells and nanolancets (see Methods). Third, sharp‐tip nanostructure protrusions are fabricated inside the microchannel by controlling the lateral etching of the micropillar. This creates a nanostructure with a sharp, pointed tip that is tall enough to pierce the cell membrane (Figure S1). The silicon substrate is then anodically bonded with borosilicate glass to create a complete microfluidic device. The inset in Figure 1b shows the silicon‐glass NEST device fabricated through a commercially available semiconductor fabrication process, highlighting the parallel microfluidic channels and sharp tip nanostructures within each microchannel.

The device geometry is optimized for each cell type. For the HeLa cells used in this study, we found the optimal channel dimensions were a height of 12 µm and a width of 8 µm. Each nanolancet consists of a single sharp tip with an approximate tip width of 100 nm, height of 2.4 µm, and length of 4 µm (Figure S1). Since there is only one nanolancet per channel, in the case of unidirectional flow, only one pore can be produced per cell.

Device operation parameters also vary by cell type. For HeLa cells, we found the optimal inlet pressure to be 28 psi [17], which results in an approximate flow speed of 3000 mm/s at the nanolancet. At 28 psi, the device can process 200 000 cells in under 2 s. All findings reported here use the optimal operational conditions noted above.

### 2.2 Quantification of Pore Generation

**FIGURE 2:**
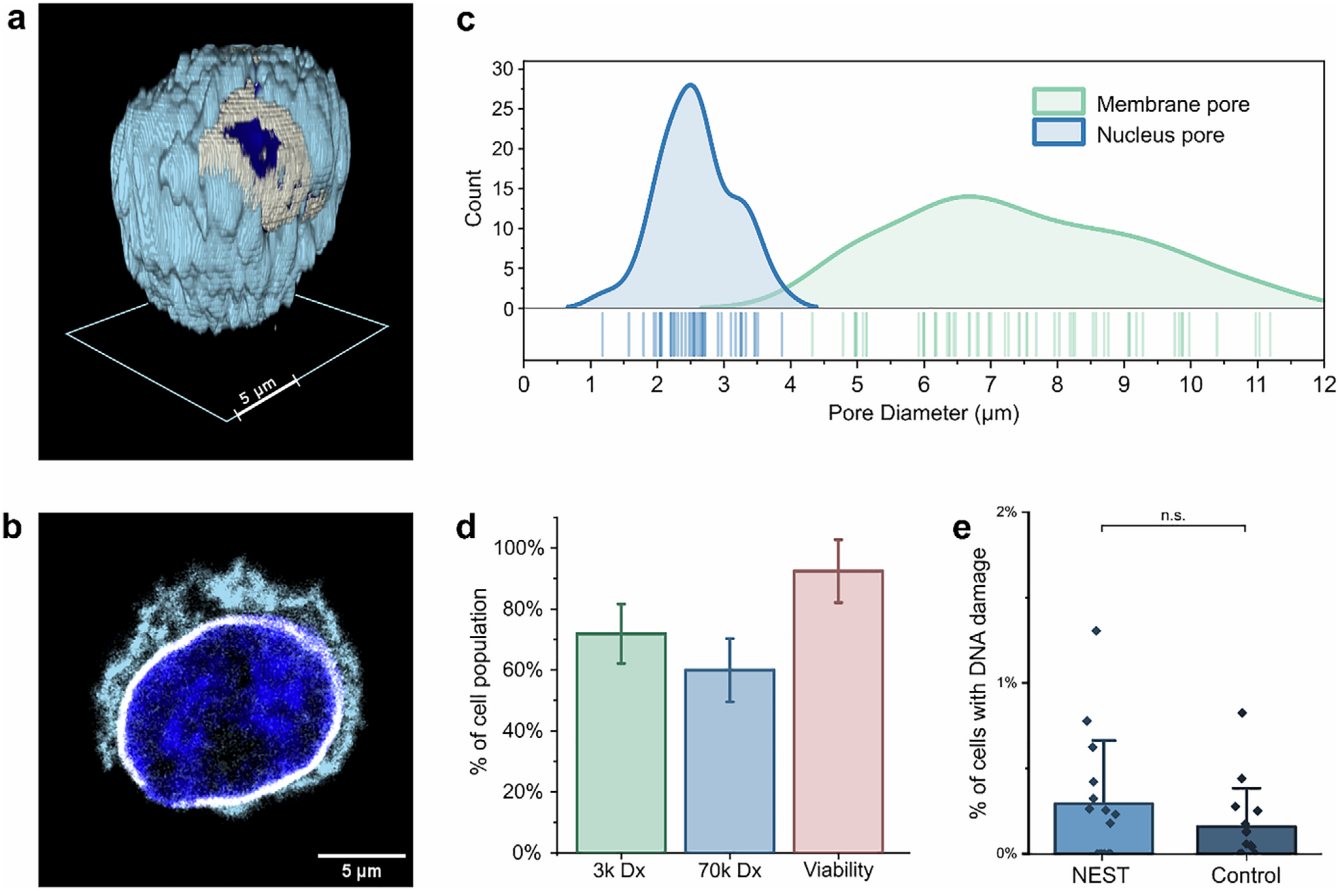
Assessments of pore generation and cell recovery after NEST treatment. (a) 3D reconstruction of confocal images of a representative HeLa cell after NEST treatment. (b) A confocal micrograph showing a slice of the cell in panel A, where discontinuities in the cell membrane and nuclear envelope are apparent. Lamin A/C (white) for the nuclear envelope, Hoechst 33324 (dark blue) for the nuclear contents, MemBrite kit (light blue) for the plasma membrane. (c) Histogram with kernel smoothing applied showing the distribution of cell membrane and nuclear envelope pore diameters, measured at the widest point of the pore visible by confocal microscopy. For the cell membrane pores, *n* = 59; for the nuclear envelope pores, *n* = 43. (d) Delivery of fluorescently‐labeled small (3 kDa Dx) and large (70 kDa Dx) dextran to HeLa cells from NEST treatment, demonstration pore generation in about 60% of the cell sample. Viability is measured by propidium iodide uptake after cell recovery immediately following NEST treatment, with PI‐positive cells considered non‐viable, showing about 90% recovery of the cell sample. *n* = 4 replicates, error bars indicate s.d. Example gating schemes are found in Figure S7. (e) Quantification of DNA damage in a NEST‐treated sample vs. an untreated control, measured by fluorescent imaging of pH2AX histone recruitment. No significant difference is found between the two sample types (student's two‐tailed t‐test, equal variance, *p* = 0.24; error bars indicate s.d.). Note: images in A and B have been recolored from the original fluorophore colors for clarity. A has been adjusted for brightness and smoothed to clarify the shape of the cell and enhance visibility of the pore. The unaltered images can be found in Figure S2.

Reproducible and synchronous poration of cells can enable single‐cell studies of recovery dynamics. To demonstrate rupture of both the cell membrane and nuclear envelope, we performed confocal laser scanning microscopy. HeLa cells were stained to show the nucleus, cell membrane, then flowed through the NEST device using a portable and power‐free syringe pump [21], and finally stained to show the nuclear envelope. Confocal imaging revealed pores in both the cell and nuclear membranes (Figure 2a,b). The average diameter of the cell membrane pore was 7.4 µm (SD = 1.8 µm, *n* = 59), and the average diameter of the nucleus pore was 2.6 µm (SD = 0.6 µm, *n* = 43), as measured using confocal microscopy images in Fiji ImageJ software (Figure 2c). Previous reports show that the NEST device allows delivery of medium‐sized (70 kDa) dextran into the nuclear space [17]. To measure the percentage of the cell population whose membranes are disrupted by the NEST device, we added 3 and 70 kDa fluorescently‐labeled dextran to the flow buffer surrounding the cells during NEST treatment. The cells were collected after treatment, allowed to recover, then analyzed via flow cytometry. Propidium iodide was added immediately before flow cytometry to measure cell viability. The flow cytometry results show a high percentage of cells with disrupted membranes, as evidenced by the delivery of both small 3 kDa dextran and large 70 kDa dextran (Figure 2d). The majority of cells recover from NEST treatment, as shown by the approximately 90% viability after treatment and recovery (Figure 2d).

**FIGURE 3:**
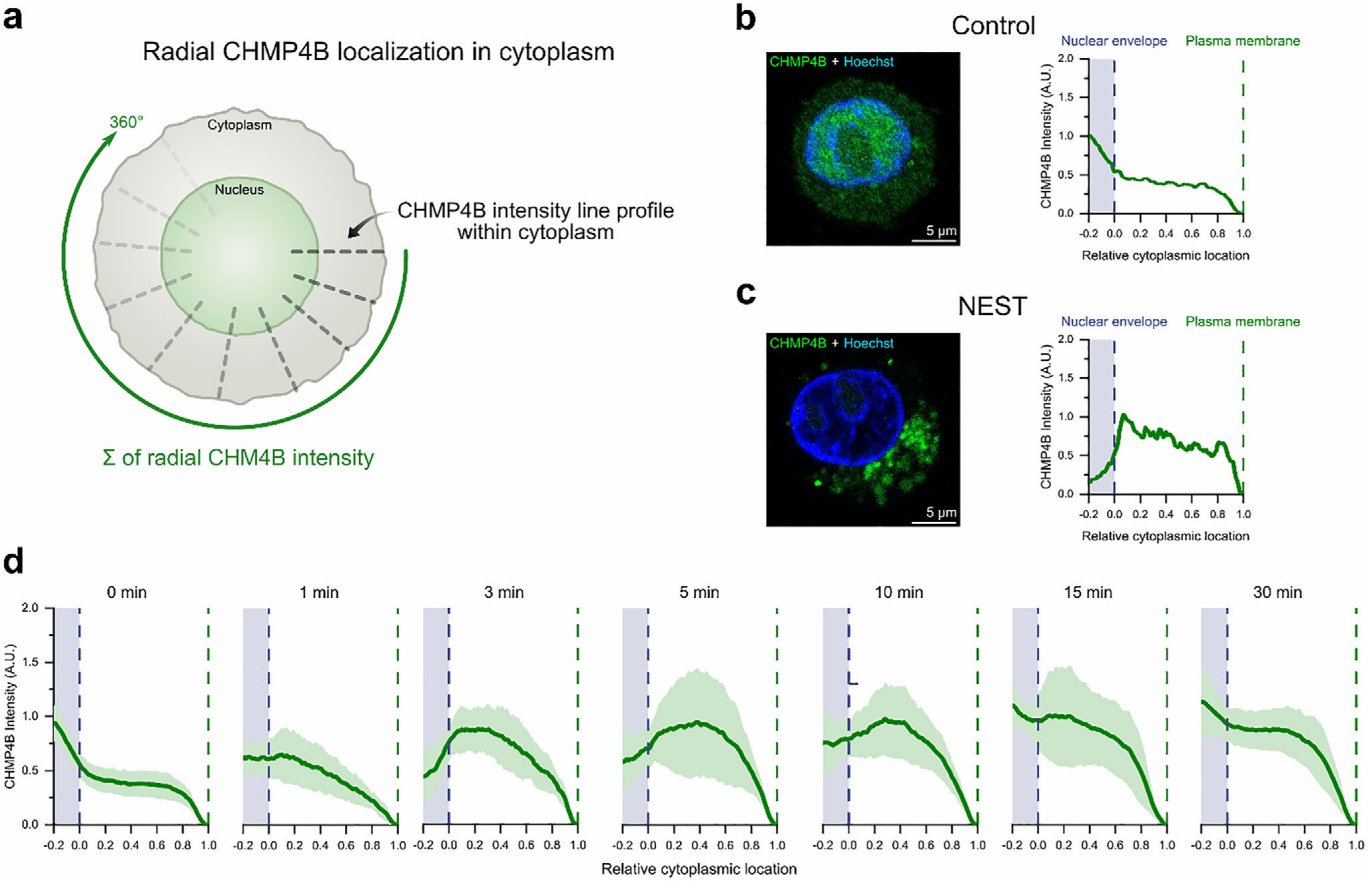
Intracellular distribution analysis of CHMP4B, an ESCRT‐III associating protein, reveals cell membrane rupture and recovery dynamics following NEST treatment. (a), Schematic representation of CHMP4B‐GFP fluorescence intensity profile analysis. The fluorescence intensity of CHMP4B was measured during a 360° rotation, consisting of 180 line profiles spanning from the nuclear envelope to the cell plasma membrane. (b), Confocal microscopy image of untreated HeLa CHMP4B‐GFP cells and corresponding radial distribution of CHMP4B‐GFP. The purple and green dashed lines represent the nuclear envelope and plasma membrane, respectively. The shaded purple area indicates the nucleoplasm (x = −0.2–0.0), while the white area indicates the cytoplasm (x = 0.0–1.0). CHMP4B proteins exhibit a dispersed distribution throughout the entire cell, with higher concentrations in the nucleus. (c), Confocal microscopy image of NEST‐treated CHMP4B‐GFP HeLa cells with the CHMP4B‐GFP radial distribution profile. CHMP4B proteins aggregate and localize around the boundary between the nuclear envelope and cytoplasmic space (purple dashed line). (d), Temporal dynamics of CHMP4B‐GFP protein profiles at time points 0, 1, 3, 5, 10, 15, and 30 min after recovery from NEST treatment. The shaded green areas indicate s.d. (*n* = 12–15).

**FIGURE 4:**
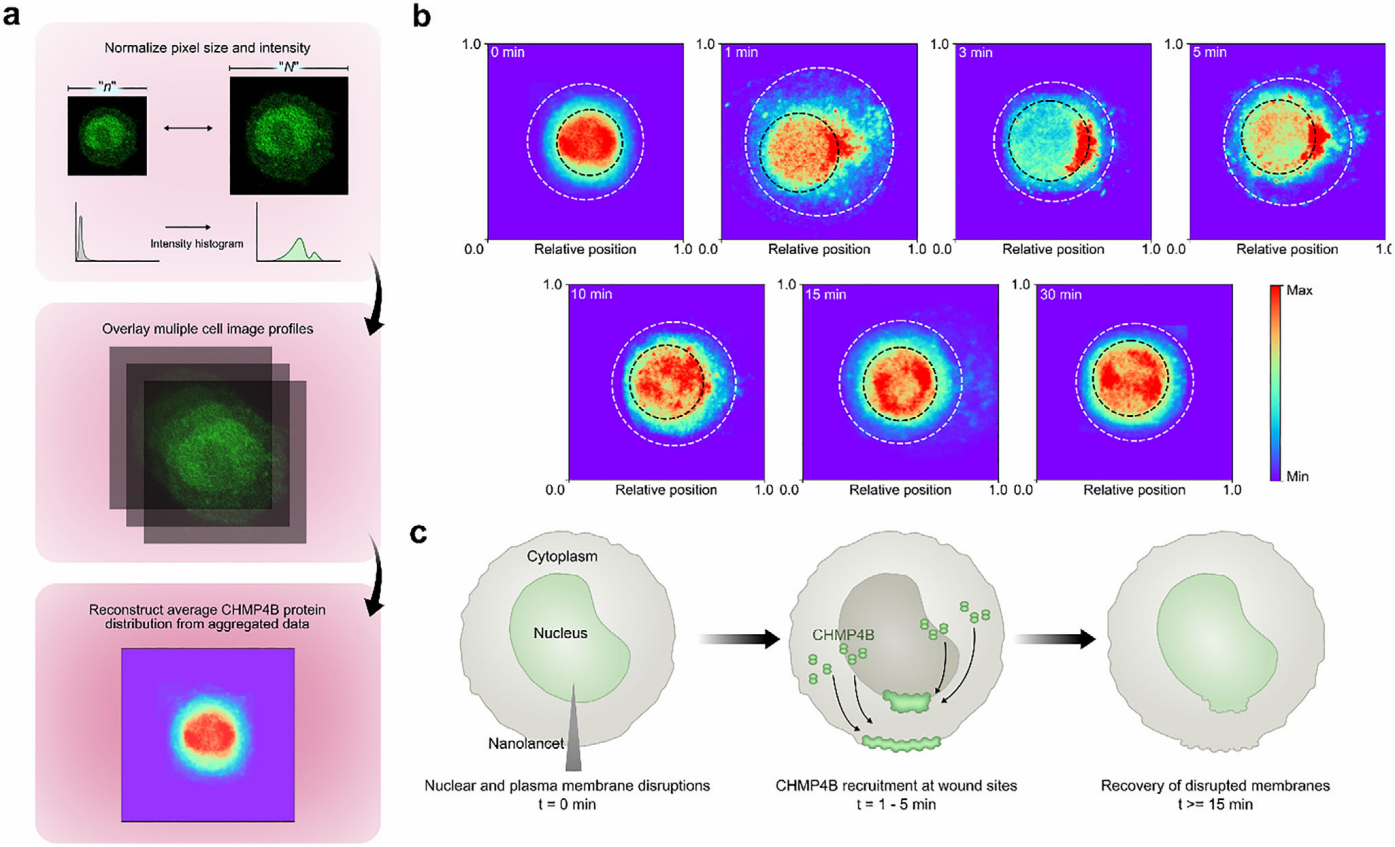
Spatial mapping of CHMP4B‐GFP aggregation confirmed its intracellular localization on the nuclear envelope. (A), Image processing workflow to analyze the 2D spatial distribution profile of the CHMP4B protein. (B), Mean distribution profile of CHMP4B protein at time point 0, 1, 3, 5, 10, 15, and 30 min after recovery from NEST treatment. White and black dashed lines represent the estimated average of cell plasma membrane and nuclear envelope size respectively. (C), Hypothesized mechanism of plasma membrane and nuclear envelope rupture triggered by the NEST device. CHMP4B proteins' initial intracellular distribution is dispersed in both the cytoplasm and nucleus. After NEST treatment, CHMP4B is recruited to the wound site on the nuclear envelope and plasma membrane, leaving the nucleus. After full repair of the wounded area, CHMP4B returns to the nucleus.

Finally, to assess DNA damage as a result of NEST treatment, we measured the recruitment of histone pH2AX to cell nuclei, a known indicator of double‐stranded DNA breaks, using a commercially available kit. The NEST‐treated cells showed no statistically significant difference in histone recruitment when compared to untreated control cells (means 0.292% and 0.158% respectively, p = 0.242, Cohen's d = 0.44), suggesting that the NEST device does not induce significant DNA damage in the studied conditions. Assessments of cell proliferation after NEST treatment show no significant changes after treatment [17]. It is possible that the rapid ESCRT‐III‐mediated repair processes described in Figures 3 and 4 limit exposure of the nuclear contents to the cytosol, thereby limited DNA damage and recruitment of DNA repair machinery. However, given the modest effect size of DNA damage, further studies with increased replicates are warranted to fully exclude damage to cellular DNA (Figure 2e).

### 2.3 ESCRT Protein Localization Analyses Reveal Membrane Recovery Dynamics

Mechanoporation by the NEST nanolancet produces large pores in the cell membrane and the nuclear envelope (Figure 2a,b), from which the cell can effectively recover. In general, the integrity of the plasma membrane and nuclear envelope is maintained by ESCRT‐III, which is recruited to the site of membrane injury as part of the cell repair mechanism [9, 10, 12]. Due to its extensive role in membrane reorganization, monitoring the distribution of ESCRT‐III proteins can reveal the dynamics of membrane wound repair mechanisms. To achieve this, we used HeLa cells that stably express GFP‐tagged charged multivesicular body protein 4B (CHMP4B) protein, a component of the ESCRT‐III complex (Figure 3, Figure S3). We performed intracellular distribution analysis of the CHMP4B proteins following NEST treatment (Figure 3a). CHMP4B‐GFP fluorescence intensity was analyzed with respect to its relative distance between the nuclear envelope to the plasma membrane [22]. 180 line profiles were drawn on confocal fluorescent micrographs to measure the CHMP4B‐GFP fluorescence intensity profile across the cytoplasm. The measured CHMP4B‐GFP intensity profiles were combined for single‐cell radial distribution profiles, shown in Figure 3 at various fixation timepoints after NEST treatment.

In untreated HeLa CHMP4B‐GFP cells, CHMP4B proteins were diffusely distributed throughout the cytoplasm with the predominant concentration of proteins found in the nucleus (Figure 3b, Figure S3a–c), consistent with previous reports [23, 24]. After NEST treatment, CHMP4B proteins were recruited to the wound site and localized to the cytoplasmic space between the nucleus and plasma membrane (Figure 3c), resulting in a depletion of CHMP4B in the nucleus. We compared the relative intensity of CHMP4B proteins between the localization site and the nucleus, revealing that the maximum enrichment of CHMP4B at the localization site was up to 10 times greater than that in the nucleus (Figure S3b), resulting from a combination of CHMP4B aggregation at the wound site and the depletion of CHMP4B from the nuclear surface. 3D volume reconstruction of the confocal images confirmed that the aggregation of CHMP4B occurred only at a single localized area on the nuclear envelope surface (Figure S4). Previous studies have shown that small wounds at the nuclear envelope trigger CHMP4B protein recruitment at the rupture site while still maintaining CHMP4B distribution in the nucleus [9, 12]. The extreme mechanical damage caused by the NEST device demonstrates that the cellular repair response scales with the severity of damage, showing that major rupture of the nuclear envelope and plasma membrane can cause a relative depletion of CHMP4B proteins in the nucleus.

We analyzed the dynamics of the CHMP4B radial distribution at different time points after treatment in 12–15 cells per timepoint for a total of 97 cells analyzed (Figure 3d). As confirmed through microscopy images, CHMP4B intensity was higher in the nucleus compared to the cytoplasm before membrane rupture (time = 0 min). Following NEST treatment to the HeLa CHMP4B‐GFP cells, the major recruitment events at the wound site occurred between 1–5 min. After 10 min, the CHMP4B aggregation dissipated, and the CHMP4B distribution profile showed higher intensity inside the nucleus, similar to the state prior to NEST treatment (Figure S5).

We further elucidated the spatial distribution profile of the CHMP4B protein by analyzing multiple confocal fluorescence images of the cells after NEST treatment (Figure 4). Figure 4a shows the workflow used to analyze the CHMP4B profile of multiple cell samples. We first normalized the size and fluorescence intensity distribution of each image, which captured a single cell per image. Then, images were rotated to align the apparent wound sites, then translated horizontally to align the left edge of each nucleus (see Methods for a detailed description of image analysis procedures). Finally, the images were overlayed to create the heatmaps shown in Figure 4, with the average cell and nuclear perimeter boundaries overlayed as dotted lines on each heatmap.

The mapped images show the average distribution of CHMP4B within the cell at time points 0, 1, 3, 5, 10, 15, and 30 min after NEST treatment (Figure 4b). In untreated cells (0 min), mapping of the average CHMP4B distribution indicates a high probability of CHMP4B protein localization within the nuclear perimeter. At time points 1–5 min, a significant portion of the visible CHMP4B appears localized to a region along the nuclear perimeter. After 10 min, the localized distribution of CHMP4B begins to dissipate and return to the inside of the nuclear perimeter.

Mapping the average CHMP4B distribution profile suggests that the NEST device creates a precise wound at the nuclear envelope where cellular repair proteins are recruited (Figure 4c). The mechanoporation of the nuclear envelope induced by NEST has completely different wound repair dynamics compared to small lesions, where the CHMP4B protein generally appears near the wound site in smaller aggregates next to the nuclear envelope [9, 12]. Here, however, the aggregation of the CHMP4B protein extends from the nucleus boundary all the way to the plasma membrane, spanning the cytoplasm, with a reduced accumulation of CHMP4B protein inside the nucleus (Figure 3c). The large‐scale damage induced by the NEST device enables a CHMP4B response of greater magnitude than small wounds. Moreover, this aggregation of the CHMP4B protein is apparently more concentrated at the area closer to the nuclear envelope (Figure 4b), indicating repair of the nuclear envelope wound, which is also supported by confocal microscopy images showing the nuclear envelope rupture in conjunction with CHMP4B localization (Figure S6).

## 3 Discussion

In this study, we found that extreme wounds on the cell membrane and nuclear envelope trigger a massive ESCRT response, characterized by trafficking of CHMP4B out of the nucleus and to the site of membrane damage, with apparent prioritization of nuclear envelope repair. The CHMP4B aggregation pattern we observed, characterized by a depletion of CHMP4B in the nucleus, is distinct from prior publications studying repair responses to small wounds [10]. The depletion of CHMP4B from the nucleus suggests the existence of a wound severity threshold beyond which the cell must prioritize repair resources, potentially limiting the capacity for repair of multiple membrane systems or for very large cellular wounds. Our findings also suggest preferential allocation of resources toward nuclear envelope repair under extreme damage to both the plasma membrane and nuclear envelope. This may be due to the complexity of the nuclear envelope compared to the plasma membrane; the nuclear envelope consists of both an inner and outer membrane as well as a network of lamins that provide structure and rigidity to the nucleus, while the plasma membrane consists of a single lipid bilayer. The apparent repair priority may also be related to the importance of the nuclear envelope in maintaining genomic integrity.

ESCRT‐III machinery plays various roles in membrane dynamics, such as plasma membrane and nuclear envelope repair and organelle compartmentalization [25, 26, 27, 28, 29]. At the membrane boundary, ESCRT protein activity can occur on both the cytoplasmic and nucleoplasmic sides of the nuclear envelope [30, 31]. After mitosis, ESCRT is recruited by LEM2, an inner nuclear membrane protein, to seal the nuclear envelope fissure [32, 33, 34]. Mechanical stress during cell migration may cause nuclear envelope rupture, triggering ESCRT recruitment at the rupture site due to the interaction of barrier‐to‐autointegration factor (BAF), exposed chromatin, and LEM proteins [35]. The active domain for ESCRT translocation is likely dependent on the type of rupture and assembly required to seal the membranes. Using NEST treatment to induce large ruptures in the plasma membrane and nuclear envelope, we were able to observe the activity of CHMP4B protein translocation dynamics. During repair, the CHMP4B proteins mainly aggregated on the cytosolic side of the rupture (Figure S5). There was no significant activity of the CHMP4B proteins from within the nucleoplasmic space. Furthermore, most of the CHMP4B residing in the nucleus translocated to the cytoplasm to repair the nucleoplasmic wound generated by the NEST device.

Existing tools to generate membrane damage and initiate repair processes predominantly generate small pores, biasing findings on cell repair mechanisms toward small wound responses. However, large ruptures have physiological relevance in cancer cell migration. Our findings showing preferential allocation of ESCRT machinery to nuclear envelope damage is severity‐dependent and only visible for extreme wounds where the scale of damage approaches the limit of what can be repaired. In this situation, the cell must choose to allocate resources where they are needed most, and these resource allocation decisions would not be visible without the large wounds generated by NEST. Beyond repair dynamics, the ability to generate controlled, yet severe cellular wounds in high throughput opens new avenues for studying the threshold between cell repair and death. The cellular decisions that are involved in initiating repair processes or death processes are an important topic of investigation for tissue injury and disease that could lead to new drugs and therapies [36, 37, 38, 39, 40]. The NEST device can help determine the threshold of irreversible damage to the cell that induces necrosis, or the threshold at which cells transition to other forms of regulated death.

The synchronous, high‐throughput wounding capabilities of NEST complement existing single‐cell wounding approaches. Because wounding of the cells is synchronized, analysis of cells fixed at various timepoints after treatment provides a statistical method to study repair dynamics across thousands of cells simultaneously, an approach that is inaccessible using traditional single‐cell wounding techniques. This population‐scale approach also enables detection of rare repair phenomena and heterogenous responses that only occur in a small portion of damaged cells, as well as weak signals that are undetectable on the scale of tens or hundreds of cells. Live cell imaging approaches, such as AFM‐ or micropipette‐based wounding, allow for superior temporal resolution for tracking individual cell responses, and future studies combining the population‐scale synchronization of NEST with live imaging of representative cell subsets would further advance our understanding of cellular repair dynamics.

As with any platform, the NEST device also presents limitations. The high‐throughput nature of this approach represents a tradeoff between throughput and temporal resolution. To address this limitation, we fixed cells at varying timepoints after NEST treatment. However, this approach does not allow for continuous evaluation of cell responses after wounding, and our findings would be complemented by live imaging studies. Although the CHMP4B imaging analysis used approximately 12–15 cells per timepoint, the critical features of NEST at play are the large pores generated and synchronous wounding across the entire population of cells. The throughput advantages of NEST are most directly represented in the population‐level flow cytometric assay and the DNA damage analyses, both performed on samples of tens of thousands of cells (Figure 2d,e, respectively). The high‐throughput capabilities of this platform may find utility in future studies, where they can be used to study population‐level responses to synchronous cell wounding.

Our approach to image analysis involved cell‐size normalization, which allows for population‐level heatmap analysis, but removed information on rupture size. To overcome this, we characterized rupture size separately by confocal microscopy. Additionally, these studies were performed in HeLa cells, and the generalizability of our findings to primary cells and diseased cell models merits further investigation. Our findings focused on CHMP4B as a representative member of the ESCRT‐III complex, but the behavior of other ESCRT machinery under severe mechanical rupture requires future investigation.

The NEST device provides a platform to investigate severe, synchronous wounding of the cell membrane and nuclear envelope in large cell populations. Our findings demonstrate that repair responses scale with wound severity, a previously unexplored aspect of cellular repair, with apparently prioritization of nuclear envelope repair under extreme damage. These severity‐dependent repair dynamics advance our understanding of the cellular response to extreme damage and have direct relevance to cancer biology, where nuclear envelope rupture during confined migration can influence genomic stability and metastatic potential. Looking forward, the NEST platform may find utility in studies of laminopathic disease models and in the molecular mechanisms that govern the transition from cell repair to regulated cell death in a previously inaccessible regime of cell damage.

## 4 Materials and Methods

### 4.1 Device Fabrication

The NEST device was fabricated using a combination of techniques, including photolithography, deep reactive ion etching (DRIE), oxide growth, and oxide removal. To create the device, a 4 inch silicon wafer (El‐Cat Inc., Ridgefield Park, NJ) was initially cleaned with O2 plasma and spin coated with photoresist Microposit S1813 (Kayaku Advanced Materials, Inc, Westborough, MA). Subsequently, the wafer was exposed to UV light (EVG EV‐420, St. Florian am Inn, Austria) and developed in MF‐26A developer (Kayaku Advanced Materials, Inc, Westborough, MA). To create deep trenches of similar height to the cell diameter, the wafer underwent the DRIE Bosch process (Oxford Instruments Plasmalab 100 ICP, Concord, MA). The target width of the microchannels was between 50%–80% of the cell diameter. Sharp tip nanostructures were generated by growing silicon dioxide on the etched wafer (ProTemp Diffusion Furnace, Santa Clara, CA), followed by etching the oxide layer using BOE 6:1. Inlet and outlet access holes were then created using laser cutting (ULS‐25, Universal Laser Systems, Inc., Scottsdale, AZ) on a Borofloat 33 wafer (UniversityWafer, Inc., South Boston, MA). The silicon wafer with micro/nanostructures and the borofloat wafer with inlet/outlet holes were aligned based on the inlet/outlet locations, then anodically bonded (EVG 520 IS, St. Florian am Inn, Austria). Finally, bonded wafers were diced (DAD3220, Tokyo, Japan) into rectangular shapes to create individual devices.

### 4.2 Cell Culture

HeLa cells (HeLa S3 and CCL‐2) were generously gifted by the Prof. Xuedong Liu's lab at the University of Colorado Boulder. To culture the cells, a T25 flask (FB012935, Fisher Scientific, Hampton, NH) with 5 mL of growth medium was used. The growth medium consisted of DMEM (Gibco 10566024, Thermo Fisher Scientific, Waltham, MA), 10% Fetal Bovine Serum (Gibco A3160602, Thermo Fisher Scientific, Waltham, MA), and 1% Penicillin‐Streptomycin (Gibco 15140122, Thermo Fisher Scientific, Waltham, MA). The HeLa CHMP4B‐GFP was gifted by Dr. Martin P. Stewart and maintained in the same growth medium but supplemented with 1% geneticin (Gibco 10131035, Thermo Fisher Scientific, Waltham, MA). The cells were passaged every 2–3 days, ensuring maximum confluency at around 80%. The cultured cells were grown in a humid environment at 37°C with 5% CO_2_.

### 4.3 Fluorescent Imaging of Plasma Membrane and Nuclear Envelope Wounds

HeLa CHMP4B‐GFP cells, a gift from Dr. Martin P. Stewart, were stained with Hoechst 33342 (Invitrogen R37605, Thermo Fisher Scientific, Waltham, MA) to observe the nucleus. The cells were harvested, suspended in DPBS, then stained with the MemBrite Fix 594/615 Cell Surface Staining Kit (30096, Biotium, Inc., Fremont, CA) per the manufacturer's instructions.

Following membrane staining, the cells were suspended in a flow buffer consisting of DPBS supplemented with 0.1% F‐68 Pluronic and 2 mm EDTA. Cells were then passed through the NEST device at an inlet pressure of 28 PSI and collected, then allowed to recover in antibiotic‐free, warm growth medium. Cells were fixed at different recovery time points (0, 1, 3, 5, 10, and 15 min) using a mixture of 2% paraformaldehyde and 1.5% glyoxal. The glyoxal solution was prepared as a 4% solution as indicated in Richter et al., 2018 [41]. Then, 3 mL of the glyoxal solution was mixed with 16% PFA in DPBS (1 mL) and DPBS (2 mL), producing 6 mL of a fixative with 2.67% PFA and 2% glyoxal. The fixative was diluted to a final concentration of 2% PFA and 1.5% glyoxal by mixing the cell sample (100 µL) with the fixative solution (300 µL). The cells were fixed at various time points after NEST treatment to capture different stages of recovery: 0, 1, 3, 5, 10, 15, and 30 min, although no significant difference in pore diameter was found at different recovery time points. The cells were fixed for 15 min at room temperature, then washed twice with DPBS and permeabilized with 0.1% triton X‐100. Nuclear envelope staining was performed using a lamin A/C mouse monoclonal antibody conjugated with AlexaFluor 647 (41357, Cell Signaling Technology, Inc., Danvers, MA) diluted 1:50 in a solution of DPBS with 0.5% BSA. The cells were stained for 1 h at room temperature, then rinsed twice with DPBS and stored under refrigeration until confocal microscopy.

The stained HeLa cells were plated in a black‐framed 96‐well plate (P96‐1.5P, Cellvis, Mountain View, CA) and centrifuged at 200 rcf for 2 min to bring the cells to the bottom of the well. A laser scanning confocal microscope (Nikon A1, Melville, NY) equipped with a 60× oil immersion objective was used to capture images and Z‐stacks of individual HeLa cells, using laser wavelengths appropriate for the fluorescent cell stains used. Z‐stacks were collected at a total height set above and below the lowest and highest visible cell membrane areas, respectively, with 0.5 εm between steps to capture a complete Z‐stack of each cell.

Confocal fluorescence images were analyzed using Fiji ImageJ (Version 1.53c) to measure the size of the cell membrane and nuclear envelope pores. First, Z‐stacks were inspected to identify the slice with the largest visible pore diameter. Then, the diameter of the cell membrane and nucleus pore were measured as the shortest distance between free edges.

### 4.4 Dextran Delivery and Flow Cytometric Analysis

HeLa CCL‐2 cells were cultured, harvested, and suspended in a flow buffer consisting of DPBS with 0.1% F‐68 Pluronic (Gibco 24040032, Thermo Fisher Scientific, Waltham, MA) and 2 mm EDTA to chelate free calcium ions. 3 kDa (Invitrogen D7132, Thermo Fisher Scientific, Waltham, MA) and 70 kDa fluorescently labeled dextrans (Invitrogen D1823, Thermo Fisher Scientific, Waltham, MA) were added to the cell suspension immediately prior to NEST treatment at a concentration of 0.1 mg/mL. The cell mixtures were then loaded into the NEST device and flowed through using a pressure‐driven syringe pump at an inlet pressure of 28 PSI. Then, the cells were collected and allowed to recover in antibiotic‐free growth medium at 37°C for 15 min. Finally, the cells were washed twice the DPBS before flow cytometric analysis.

Samples were analyzed by multicolor flow cytometry (BD FACSCelesta Cell Analyzer, BD Biosciences, Franklin Lakes, NJ). A 405 nm laser with 450/40 filter was used to detect Cascade Blue‐labeled 3kDa dextran, and a 488 nm laser with a 530/30 filter was used to detect FITC‐labeled 70 kDa dextran. Immediately prior to flow cytometry, propidium iodide (P4170, Sigma–Aldrich, Saint Louis, MO) was added to samples at a concentration of 1 µg/mL as a viability indicator, then analyzed using a 561 nm laser with 610/20 filter. Cells positive for PI were considered non‐viable. A sample gating scheme is shown in Figure S7, and all data were gated in BD FACSDiva Software (BD Biosciences). Each data point consists of a sample of 10 000 cells.

### 4.5 DNA Damage Quantification

DNA damage was assessed using a commercially available kit that allows for measurement of pH2AX histone recruitment to double‐stranded DNA breaks (Invitrogen H10292, Thermo Fisher Scientific, Waltham, MA), with the omission of the reagent to detect membrane permeability. This reagent fluoresces at the same wavelength at the FITC‐CHMP4B in the cell line used. HeLa cells were harvested, suspended in a flow buffer consisting of DPBS with 0.1% F‐68 Pluronic (Gibco 24040032, Thermo Fisher Scientific, Waltham, MA) and 2 mm EDTA, and passed through the NEST device at an inlet pressure of 28 PSI [17]. The cells were collected and allowed to recover in antibiotic‐free growth medium before being plated into a 96‐well plate. Control samples were harvested and suspended in flow buffer in parallel to the NEST‐treated samples, but were not passed through the NEST device. Similarly, the control samples were suspended in antibiotic‐free growth medium for the same duration as the NEST‐treated cells. All the cells, both the treated samples and control samples, were then cultured for 20 h under standard culture conditions (a humidified incubator with 5% CO2), before fixation and staining according to the kit manufacturer's protocol. The samples were imaged using an epifluorescence inverted microscope (Nikon Eclipse Ti2 Inverted microscope, Melville, NY) using filter sets appropriate for Hoechst/DAPI and TRITC fluorescence; images were captured using a digital camera (Hamamatsu C11450 ORCA Flash‐4.0LT, Bridgewater, NJ), and images were analyzed using Fiji ImageJ.

To minimize operator bias in image analysis, all images were processed using a standardized macro that applied identical threshold parameters across all images. Briefly, the quantity of nuclei in each image was identified in the Hoechst channel by applying an intensity threshold to exclude background signal, a watershed segmentation algorithm to resolve adjacent nuclei, and a minimum size filter of 100 pixels. pH2AX‐stain‐positive nuclei were identified in the TRITC channel by applying an intensity threshold to exclude background signal and a minimum size filter of 10 pixels. Segmented objects in both channels were counted using the Analyze Particles function. The percentage of cells showing DNA damage was quantified by dividing the number of pH2AX‐positive nuclei from the total number of Hoechst‐positive nuclei per field of view. The negative control consisted of untreated cells processed in parallel. Each data point in Figure 2E represents a field of view. The total dataset represents 5 biological replicates with a total number of cells analyzed exceeding 10 000 cells for the NEST samples and 25 000 cells for the untreated samples.

### 4.6 ESCRT‐mediated Membrane Wound Repair Study

HeLa CHMP4B‐GFP cells were stained with Hoechst 33342 (Invitrogen R37605, Thermo Fisher Scientific, Waltham, MA) to observe the nucleus. The cells were harvested and resuspended in DPBS supplemented with 0.1% F‐68 Pluronic and 2 mm EDTA. The cell suspension was flowed into the NEST device at with an inlet pressure of 28 psi. After NEST treatment, the cells were incubated with antibiotic‐free warm growth medium to initiate cell recovery. Cells were fixed at different time points of recovery (0, 1, 3, 5, 10, 15, and 30 min) using 2% paraformaldehyde for 15 min at room temperature. The fixed cells were washed twice with DPBS and stored under refrigeration, protected from light, for further analysis.

The fixed, treated HeLa CHMP4B‐GFP cells were plated on a black‐framed 96‐well plate (P96‐1.5P, Cellvis, Mountain View, CA) and centrifuged at 200 rcf for 2 min to promote settling of the suspended cells to the bottom surface. A laser scanning confocal microscope (Nikon A1, Melville, NY) equipped with a 60× oil immersion objective was used to capture images and Z‐stacks of individual HeLa CHMP4B‐GFP cells. Z‐stacks of individual HeLa cells were collected as described in Section 4.3, with appropriate channels for DAPI imaging and GFP imaging, as well as brightfield image collection.

The confocal fluorescence images were analyzed using Fiji ImageJ (Version 1.53c) and Python (version 3.8.10) with the scikit‐image library (version 0.19.3) [42, 43, 44]. To simplify analysis of CHMP4B distribution, a single image from each Z‐stack was chosen to represent the cell. This slice image was chosen on the basis of fluorescent intensity in the GFP channel; the slice with the greatest GFP fluorescent intensity was chosen as the representative image.

To identify the nucleus, the DAPI channel of each image was binarized using Otsu's thresholding, and the nuclear centroid was calculated using region properties analysis from scikit‐image. The boundary of the plasma membrane was detected from binarization of brightfield images using Otsu's thresholding.

For radial CHMP4B distribution analysis, 180 line profiles with a width of 1 pixel separated by 2°, spanning 360° total, were drawn on each cell originating at the calculated nuclear centroid. Line profiles were extracted from the CHMP4B channel, using the DAPI and brightfield channels to define nuclear and cellular boundaries, respectively. The line profiles were cropped to include the span between the nuclear boundary and plasma membrane, plus an additional 20% of the cytoplasmic length extending into the nucleus. Each profile was normalized by subtracting the minimum value and dividing by the total sum of intensity. The normalized profile was then resampled to a length of 120 pixels. The intensity at each pixel was extracted for each line profile; the mean and standard deviation were calculated to produce a CHPM4B radial distribution profile for each cell. Then, the CHMP4B profiles were averaged across the 12–15 cells imaged for each timepoint.

For heatmap generation as shown in Figure 4, representative images of cells were first re‐oriented so the apparent wounded area was aligned for each cell. This was done by finding the line profile with the highest overall CHMP4B intensity, then re‐orienting the image so that the line profile was oriented at 0° (horizontal with the nuclear region on the left side of the line profile). Then, each image was rescaled so that the nucleus occupied 20% of the image as follows: a single horizontal line profile was drawn on each rotated image. The width of the nucleus along this line profile was calculated, then the rescaling factor was calculated so that the nuclear width totaled 240 pixels of the 1200 pixel‐wide final image. Both the DAPI and CHMP4B channel images were resized according to this rescaling factor, using scikit‐image's resize function. The resized images were aligned so that the left edge of each nucleus was positioned at the same vertical position in each image. Rescaled, aligned images from the cells at each timepoint were summed to produce the final heatmaps. The heatmaps were contrast‐adjusted using percentile‐based intensity rescaling. The heatmaps were smoothed with a Gaussian filter for display purposes. Nuclear and cell boundary overlays were produced by averaging the location of the nuclear perimeter as visible in the DAPI channel along the single line profile, as well as the cell boundary visible in the brightfield channel. The averages were overlayed as a Hough circle transform using scikit‐image.

### 4.7 Statistical Analysis

All data are presented as mean ± standard deviation. No outliers were excluded from any dataset. Pore diameter measurements (Figure 2c) and CHMP4B radial distribution profiles (Figures 3d and 4) are reported descriptively and were not subjected to formal statistical hypothesis testing. Pore diameters were measured from *n* = 59 plasma membrane pores and *n* = 43 nuclear envelope pores from confocal microscopy images. The CHMP4B radial distribution profiles were averaged across *n* = 12–15 cells for each time point, with a total of 97 cells analyzed across all 7 time points.

Dextran delivery and viability (Figure 2d) were assessed by flow cytometry. Each data point represents a sample of 10 000 cells for each biological replicate (*n* = 3 biological replicates). These data are presented as percentages of the gated cell population and were not subjected to statistical hypothesis testing.

For DNA damage quantification (Figure 2e), the percentage of pH2AX‐positive nuclei was compared between NEST‐treated and untreated control samples using a two‐tailed, two‐sample Student's T‐test assuming equal variances. A total of 5 biological replicates were analyzed, with >10 000 NEST‐treated cells and >25 000 untreated control cells analyzed. Effect size was calculated using Cohen's d. Significance was defined as *p* ≤ 0.05.

Pore size and DNA damage analyses were performed in Microsoft Excel. Flow cytometry data were analyzed in OriginPro (OriginLab Corporation), and all plots presented in this manuscript were prepared in OriginPro.

## Author Contributions

**X.D**. and **A.K.F**. designed research; **A.K.F**. and **L.A**. performed research; **S.E.S**., **W.T**., and **C.P.N** contributed analytic tools and related research; **A.K.F**. and **L.A**. analyzed data; **A.K.F**. and **L.A**. wrote the manuscript; **X.D**. supervised research and revised the manuscript. All authors have given approval to the final version of the manuscript.

## Funding

This research was supported by the NIH Maximizing Investigators’ Research Award (MIRA) 1R35GM142817, W. M. Keck Foundation Medical Research grant, and the University of Colorado Boulder Research & Innovation Seed Grant. We thank Martin Stewart for valuable discussion on the manuscript and his generous help with the CHMP4B‐GFP HeLa cells, which are originally from Tony Hyman's lab at the Max Planck Institute of Molecular Cell Biology and Genetics. L.A. and A.K.F. acknowledge support from the Teets Family Endowed Doctoral Fellowship. Part of the microfabrication of the devices was performed in the Utah Nanofabrication facility at the University of Utah.

## Conflicts of Interest

A patent based on the NEST technology was granted in 04/2025. Xiaoyun Ding is involved in a new startup company “Flowpoint” that translates this NEST technique.

## Supporting information




**Supporting File**: advs76561‐sup‐0001‐SuppMat.pdf.

## Data Availability

The data that supports the findings of this study are available in the supplementary material of this article.
