# Supplementary material for: High‐Throughput Mechanical Rupture of Nuclear Envelope and the Intracellular Dynamics of Massive Wound Repair
Source: Adv Sci (Weinh). 2026 Jul 14:e76561. Online ahead of print. doi: 10.1002/advs.76561 (PMC13366362; doi:10.1002/advs.76561)
Supplement: Supplementary file 1 — Supporting File: advs76561‐sup‐0001‐SuppMat.pdf. [file ADVS-9999-e76561-s001.pdf]

Supplementary Information

High-throughput mechanical rupture of nuclear envelope and the intracellular dynamics of massive wound repair

Apresio K. Fajrial,[1,a] Leyla Akh,[1,b] Stephanie E. Schneider,[a] Wei Tan,[ab] Corey P. Neu,[abd] Xiaoyun Ding.[abcd*]

[a] Paul M. Rady Department of Mechanical Engineering, University of Colorado, Boulder, CO 80309, USA.
[b] Biomedical Engineering Program, University of Colorado, Boulder, CO 80309, USA.
[c] Material Science and Engineering Program, University of Colorado, Boulder, CO 80309, USA.
[d] BioFrontiers Institute, University of Colorado, Boulder, CO 80309, USA.
[1] Authors contributed equally to this work.
* Corresponding author: Xiaoyun Ding
Email: xiaoyun.ding@colorado.edu

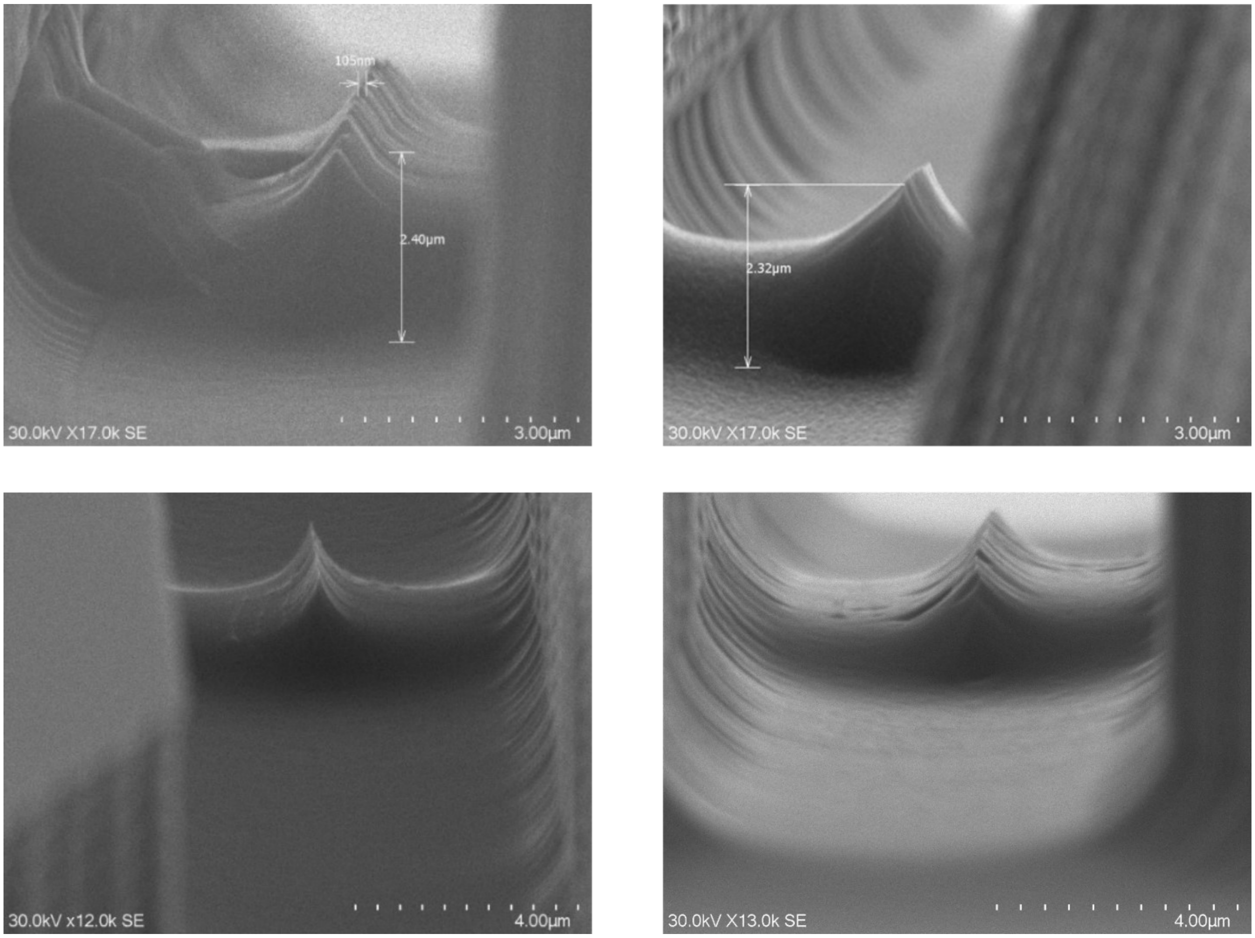

Supplementary Figure 1 | Tilted angle SEM image of four representative NEST nanolancets. Each nanolancet is comprised of one tip with a length of approximately 4 µm.

a 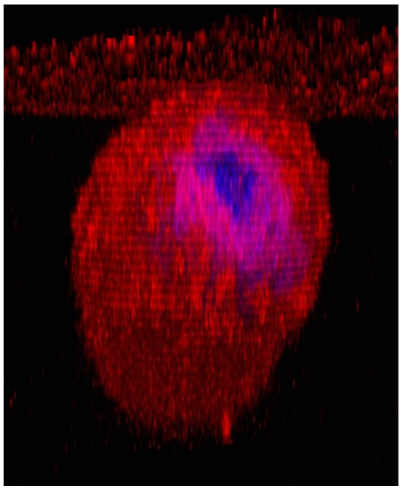 b 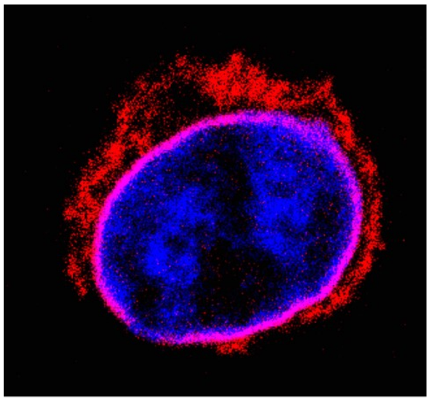

Supplementary Figure 2 | Original confocal images of pore in cells. (a) 3D reconstruction of laser-scanning confocal images of a representative cell with a visible pore in the cell membrane (red) and nuclear envelope (magenta), showing the stained DNA inside the nucleus (blue). (b) confocal micrograph showing a slice of the cell in panel A, where discontinuities in the cell membrane and nuclear envelope are apparent. These images were recolored and processed for Figure 1 in the main manuscript due to the similarity between the red and magenta fluorophores, as well as mild photobleaching visible in part of the cell membrane.

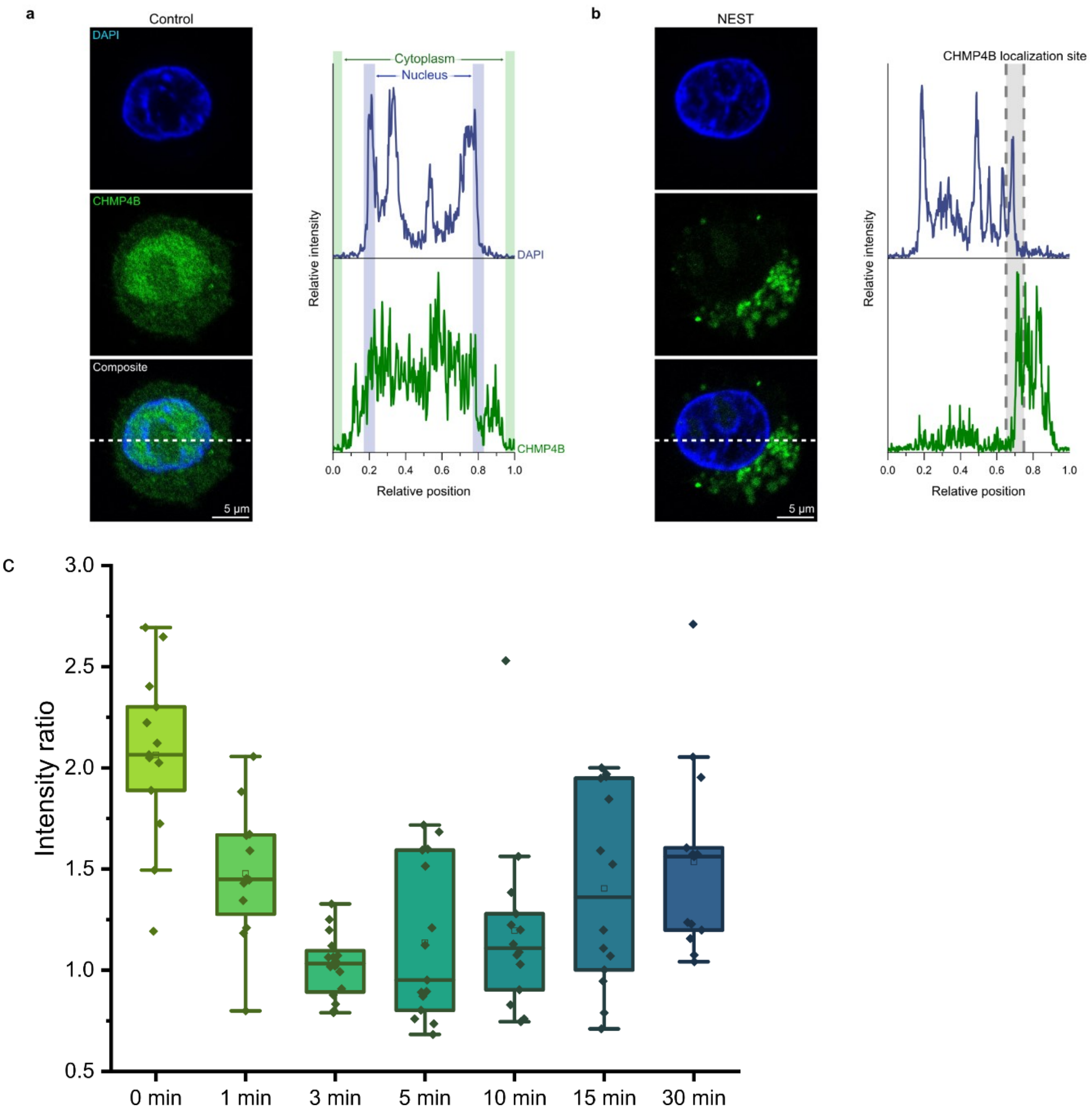

Supplementary Figure 3 | CHMP4B intensity profile from confocal fluorescence images. 3D confocal volume reconstruction of HeLa CHMP4B-GFP cells after NEST treatment. a, Confocal microscopy image of untreated CHMP4B HeLa cells and quantification of the fluorescent intensity profile. Relative positions 0.0 and 1.0 indicate two ends of the cell plasma membrane. **b**, Confocal microscopy image of NEST-treated CHMP4B HeLa cells. CHMP4B proteins aggregate and localize around the boundary between the nuclear envelop and cytoplasmic space (shaded gray region). **c**, the ratio of fluorescence intensity inside the nucleus vs in the cytoplasm at various timepoints of recovery after wounding. 180 line profiles spanning 360° around each cell image were calculated, then resampled for a total length of 100 pixels between the nuclear perimeter and plasma membrane with an additional 20 pixels extending into the nucleoplasmic space. We computed the average fluorescent intensity of CHMP4B in the nucleus and CHMP4B in the cytoplasm along each line profile, then produced a ratio of nuclear/cytoplasmic (N/C) CHMP4B intensity for each line profile. These were averaged to produce an N/C ratio for each cell. This analysis was performed for each time point, and every data point in panel C below represents the averaged N/C ratio for one cell. p-values from a two-tailed student's T test compared to the t=0 min timpoint, corrected for multiple comparisons using the Bonferroni adjustment: t =

1 min, p=0.005; t = 3 min, p = 3.5×10^{-6}; t = 5 min, p = 1.7×10^{-5}; t = 10 min, p = 0.00015; t = 15 min, p = 0.005; t = 30 min, p = 0.039.

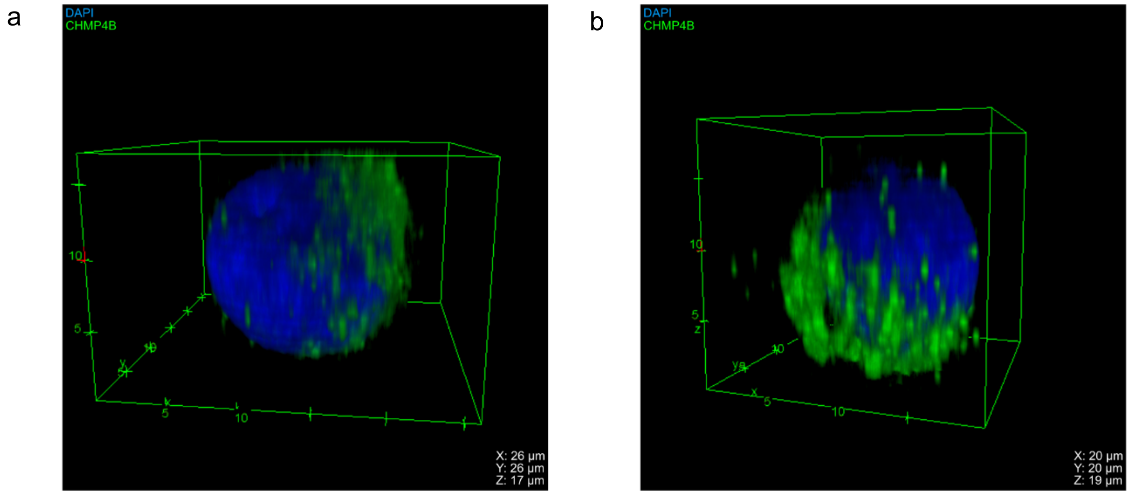

Supplementary Figure 4 | 3D confocal volume reconstruction of HeLa CHMP4B-GFP cells after NEST treatment. The left and right figures are two representative samples showing localized CHMP4B aggregation at a single concentrated area on the nuclear envelope.

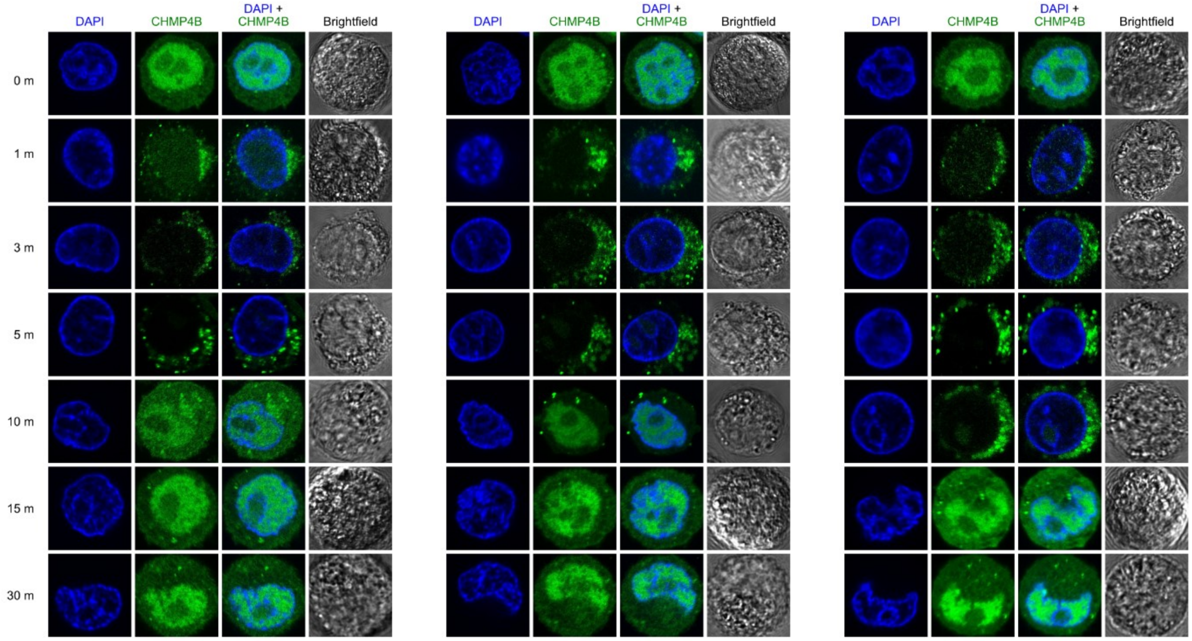

Supplementary Figure 5 | Dynamics of ESCRT-III recruitment on the nuclear envelope after NEST treatment. Representative images of HeLa cells expressing CHMP4B protein, a subunit of ESCRT-III complex, at time points 0, 1, 3, 5, 10, 15, and 30 m after NEST treatment. Large aggregates of CHMP4B proteins were localized at the nuclear surface at time points 1, 3, and 5 m. The CHMP4B protein mainly resided on the cytoplasm side of the puncture, not the nucleoplasm space. CHMP4B protein distribution returns to baseline state at time points 15 m.

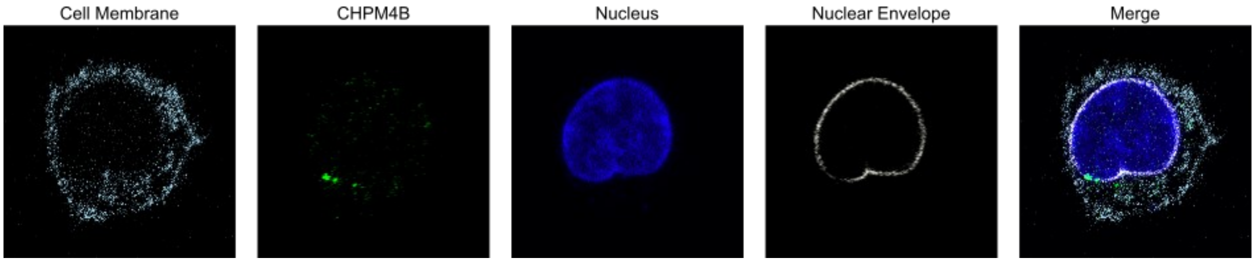

Supplementary Figure 6 | CHMP4B aggregation at the site of nuclear damage. Representative confocal microscopy images of a cell with a ruptured membrane showing CHMP4B aggregation at the site of membrane rupture.

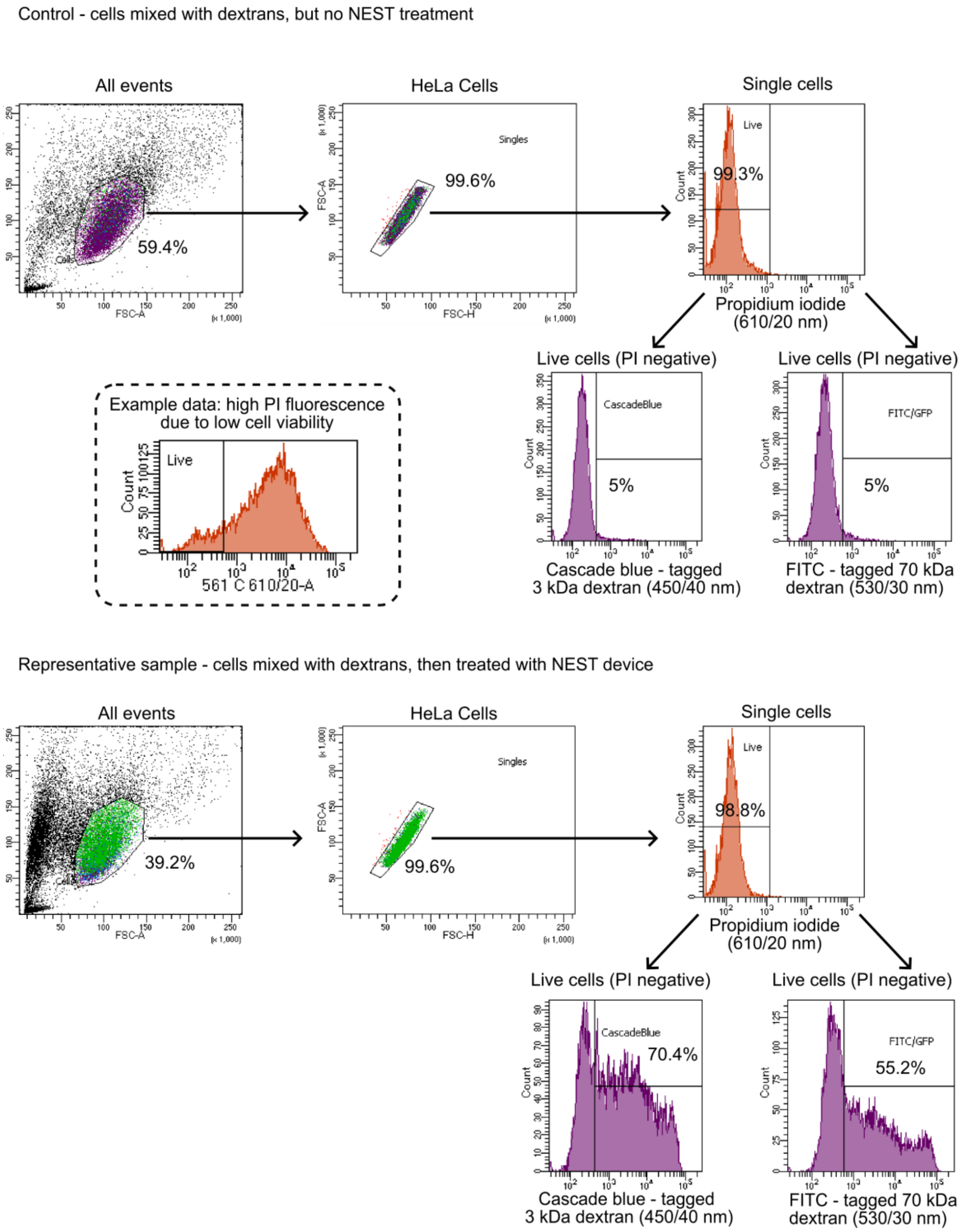

Supplementary figure 7 | flow cytometry gating scheme. Control samples treated with dextran are set at 5% fluorescence in the cascade blue and FITC gates to account for surface binding of dextrans. PI samples are gated as live if they do not show significant PI fluorescence. Inset shows a sample of data where viability is low and PI fluorescence is high. This condition was achieved using smaller device dimensions than the other results shown here and in the main text.
